# Whole genome sequencing of *Brucella melitensis* isolated from 57 patients in Germany reveals high diversity in strains from Middle East

**DOI:** 10.1371/journal.pone.0175425

**Published:** 2017-04-07

**Authors:** Enrico Georgi, Mathias C. Walter, Marie-Theres Pfalzgraf, Bernd H. Northoff, Lesca M. Holdt, Holger C. Scholz, Lothar Zoeller, Sabine Zange, Markus H. Antwerpen

**Affiliations:** 1 Bundeswehr Institute of Microbiology, Munich, Germany; 2 Institute of Laboratory Medicine, Ludwig-Maximilians University, Munich, Germany; 3 National Consultant Laboratory for *Brucella*, Munich, Germany; East Carolina University Brody School of Medicine, UNITED STATES

## Abstract

Brucellosis, a worldwide common bacterial zoonotic disease, has become quite rare in Northern and Western Europe. However, since 2014 a significant increase of imported infections caused by *Brucella (B*.*) melitensis* has been noticed in Germany. Patients predominantly originated from Middle East including Turkey and Syria. These circumstances afforded an opportunity to gain insights into the population structure of *Brucella* strains. *Brucella*-isolates from 57 patients were recovered between January 2014 and June 2016 with culture confirmed brucellosis by the National Consultant Laboratory for *Brucella*. Their whole genome sequences were generated using the Illumina MiSeq platform. A whole genome-based SNP typing assay was developed in order to resolve geographically attributed genetic clusters. Results were compared to MLVA typing results, the current gold-standard of *Brucella* typing. In addition, sequences were examined for possible genetic variation within target regions of molecular diagnostic assays. Phylogenetic analyses revealed spatial clustering and distinguished strains from different patients in either case, whereas multiple isolates from a single patient or technical replicates showed identical SNP and MLVA profiles. By including WGS data from the NCBI database, five major genotypes were identified. Notably, strains originating from Turkey showed a high diversity and grouped into seven subclusters of genotype II. MLVA analysis congruently clustered all isolates and predominantly matched the East Mediterranean genetic clade. This study confirms whole-genome based SNP-analysis as a powerful tool for accurate typing of *B*. *melitensis*. Furthermore it allows special allocation and therefore provides useful information on the geographic origin for trace-back analysis. However, the lack of reliable metadata in public databases often prevents a resolution below geographic regions or country levels and corresponding precise trace-back analysis. Once this obstacle is resolved, WGS-derived bacterial typing adds an important method to complement epidemiological surveys during outbreak investigations. This is the first report of a detailed genetic investigation of an extensive collection of *B*. *melitensis* strains isolated from human cases in Germany.

## Introduction

On a global scale human brucellosis is one of the most common bacterial zoonotic diseases [1]. However, its occurrence greatly differs between geographic areas throughout the world. The disease has its highest incidence and prevalence in countries of the Mediterranean basin, Middle East, some parts of Central and South America, Africa and Asia [2]. The highest annual incidences of human brucellosis per million of the population are observed in Syria and in Mongolia [3]. In contrast, countries of Northern and Western Europe are considered free of autochthonous human brucellosis. However, each year a relatively small number of cases are reported in Germany, with most of them having a history of travelling to or immigrating from endemic regions like the Mediterranean basin. In total, 267 cases of human brucellosis with a median number of 24 cases per annum were reported during the last decade (2005–2015) to the national surveillance system at the Robert Koch Institute in Berlin (SurvStat@RKI 2.0, https://survstat.rki.de, data as of 09/05/2016). Notably, within the last two years an increase of cases was observed. In 2014 and 2015, the number of annual cases nearly doubled to 47 and 44, respectively.

The most important route of transmission is the ingestion of contaminated raw milk and other unpasteurized dairy products, but the disease may also be acquired by handling infected animals, animal discharges, or cultures of the pathogen [4]. Human-to-human transmissions are rare events. Clinical manifestations of brucellosis are versatile and comprise acute systemic infections with undulating fever as cardinal symptom as well as localized inflammatory processes and site-specific manifestations in chronically infected patients. A 6-week regimen comprising rifampin and doxycycline is commonly applied in Germany to eradicate the pathogen [5]. Patients with complications often need longer treatment and additional antimicrobial substances like aminoglycosides [6].

Human brucellosis can be caused by various *Brucella* species. The genus currently comprises 12 validly published species which are genetically highly related to each other [7,8]. *Brucella melitensis* is by far the most frequently observed causative agent of human infection. Based on DNA-DNA hybridization studies that revealed DNA-DNA homologies of >80%, all classical species might be attributed to a single species with several biovars [9]. However, due to historic reasons, species-specific predilections for particular animal hosts, biochemical features etc. the Subcommittee on the taxonomy of *Brucella* agreed in 2003 on a return to the pre-1986 taxonomic opinion with a six species concept (*B*. *abortus*, *B*. *canis*, *B*. *melitensis*, *B*. *neotomae*, *B*. *ovis*, *B*. *suis*) [10].

Multiple Locus Variable Number Tandem-Repeat (VNTR) Analysis (MLVA) has become a major molecular typing method to characterize several pathogenic bacterial species. *Brucella* MLVA-16 scheme has been proven to be a valuable tool in epidemiological trace-back investigations with high discriminatory power in several studies [11–17]. Recently, a full genome SNP-based phylogenetic analysis was published and could provide another powerful tool for intraspecies discrimination of *Brucella* [18].

Increasing numbers of brucellosis cases in Germany in 2014 and 2015 triggered us to investigate the geographic origin of strains isolated in Germany. Using the National Consultant Lab´s collection of clinical isolates of *B*. *melitensis* originating from 57 patients diagnosed as brucellosis cases in Germany since 2014, our study was designed (i) to trace back the strains to their geographic origin; (ii) to compare the resolution of WGS-based SNP-typing with the standard MLVA-16 genotyping; (iii) to determine genetic variation within the genomes of the strains that could affect molecular diagnostic or typing assays using next-generation sequencing technology.

## Material and methods

### Strains

All available strains from culture-positive cases of human brucellosis that had been processed by the National Consultant Laboratory for *Brucella* and that had been identified as *B*. *melitensis* by real-time and conventional PCR methods between January 2014 and June 2016 (n = 57) were subjected to whole genome sequencing (WGS). Patient histories were evaluated for travel to or residence in countries known to be endemic for brucellosis. To enlarge the covered region of geographic origin as well as to consider genomic variation over time, six strains of cases from previous years were also added to the strain collection. To assess the reproducibility of applied methods, biological (i.e. one patient, but different isolates) and technical (i.e. one isolate, but different sequence libraries) replicates were included. Overall, isolates from 63 individual patients from 17 countries were analysed (see Table 1 and S1 Table).

**Table 1:** Summary of samples grouped by country of origin.

Country	Samples*
Afghanistan	4
Albania	1
Algeria	1
Bulgaria	1
India	1
Iran	2
Iraq	6
Italy	2
Jordan	1
Kuwait	1
Morocco	1
Romania	1
Saudi Arabia	1
Somalia	2
Syria	16
Turkey	27
Turkmenistan	1
unknown	5

*This study comprises 74 samples (including biological and technical replicates) from 63 individual patients with reference to 17 countries. Genomes were uploaded to NCBI Bioproject database (Bioproject PRJNA347914).

### Whole genome sequencing

Strains were re-cultivated from MicroBank^™^ vials by standard procedures. Pure colonies were picked and inactivated using an ethanol-based in-house protocol. Genomic DNA was isolated according to manufacturer's tissue protocol of QIAamp DNA Mini Kit (Qiagen). DNA concentration was determined by use of a Qubit^®^ 2.0 Fluorometer (Thermo Fisher Scientific) and the Qubit^®^ dsDNA high sensitivity assay kit (Thermo Fisher Scientific). Nextera^®^ XT DNA Library Preparation kit (Illumina) with an input DNA amount of 1 to 3 ng was used for library preparation. Finally, whole genome sequencing was performed on a MiSeq instrument (Illumina) with corresponding MiSeq Reagent Kit v3 (600 cycle) chemistry.

### Post-sequencing data processing

The sequenced paired-end reads were assembled with SPAdes v3.9 [19]. Even with DNA fragment sizes of about 550 bp, short reads will not assemble into a single contig for each of the two chromosomes, as the *B*. *melitensis* genome contains rRNA operons as well as insertion sequence elements. For identification of a reference sequence with the most similar nucleotide identity we randomly picked five assemblies of our sequenced strains and compared them with all nine already available complete genomes (NCBI) using blastn. *B*. *melitensis* M5-90 (GenBank accessions CP001851 and CP001852) showed the highest similarity rather than the commonly used reference strain *B*. *melitensis* 16M.

Hence, resulting contigs were aligned against *B*. *melitensis* M5-90 using blastn [20] to determine their order. Contigs with multiple coverage of the average read coverage were duplicated and inserted between the appropriate contigs to get a valid and continuous mapping to the reference sequence. Afterwards, an in-house developed Python script was used to concatenate and circularize the ordered contigs into the two chromosomes by resolving overlaps or creating gaps filled with Ns of a length estimated from the mapping. BWA [21] was subsequently used to map the raw reads to the chromosomal contigs. Finally, Pilon [22] was applied to undo mis-assemblies, polish the sequence by correcting SNPs and small InDels, as well as to close gaps. In some cases, several iterations of BWA and Pilon were necessary to close longer gaps or gaps newly introduced by undoing the mis-assemblies. The polished contigs were manually checked after each cycle, corrected if necessary and uploaded to the NCBI Bioproject database (Bioproject PRJNA347914) together with the sequenced reads. Annotation was automatically performed by the NCBI Prokaryotic Genome Annotation Pipeline [23].

### Whole genome SNP analysis

For a rapid whole genome alignment of all sequenced strains, all publicly available completed *B*. *melitensis* genomes and some incomplete genome assemblies from selected strains against the reference genome *B*. *melitensis* M5-90, we used Parsnp (parameters -c -e -u -C 1000) from the Harvest suite [24]. The HarvestTools from the same suite were used to extract the SNP positions as a VCF file (parameter -V, [24]). These positions were filtered to exclude clusters of SNPs as well as SNPs located in annotated repeat regions or rRNAs as well as predicted tandem repeats (using [25] with default parameters) by creating a BED file and using it as input to the HarvestTools to extract concatenated artificial SNP sequences as a multiple FASTA file (parameters -b <BED file> -S). This FASTA file was then analyzed with the R Analysis package ape [26]. A maximum parsimony tree was created based on the Kimura nucleotide distance [27], ACCTRAN, UPGMA clustering and 1,000 bootstrap replicates using PAUP* [28]. The R Analysis package phangorn [29] was used to determine homoplastic SNPs. The VCF file was further analysed using snpEff [30] to predict and annotate the coding effects of the SNPs found (see S1 File).

### In silico PCR

Following sequence assembly, several in silico PCRs were performed using an in-house Python script to confirm species identification as well as to look out for SNPs in genomic regions usually targeted by molecular assays. In short, *bcsp31*, a single-copy locus for identification, *IS711*, a multi-copy locus for identification, *recA* [31], a target to discriminate closely related *Ochrobactrum* from *Brucella*, BruceLadder [32], a multiplex PCR with specific band patterns after gel electrophoresis to distinguish *Brucella* species from each other, as well as *rpoB* [33], a hotspot region for rifampin resistance, were included.

### In silico MLVA

All sequences also underwent in silico MLVA with 16 loci (MLVA-16, [11,12]), a recognized tool to perform epidemiological trace-back investigations of *B*. *melitensis*. As determining repeat numbers from WGS data may be error-prone, we carefully checked each locus in respect of expected total length, internal repeat homogeneity or probability to get collapsed VNTRs during the assembly. The analysis was performed by an in-house Python script and all resulting MLVA-16 genotypes were compared to a public database with 2,215 entries of *B*. *melitensis* strains that can be assessed online (http://microbesgenotyping.i2bc.paris-saclay.fr/, [34]).

## Results

### Whole genome assemblies

The median MiSeq library output was 2x 906,000 reads (ranging from 290,252 to 2,126,996) resulting in an average coverage of 75-fold (27–159). De novo assembly using SPAdes ended in about 21 initial contigs (14–31) for each strain. Ordered scaffolding and polishing using two iterations of Pilon resulted in continuous chromosomes with an average of three gaps mostly located in tandem repeat or homopolymer regions summing up to 200 missing bases. None of the gaps were located in MLVA marker regions. The genome of five strains resulted in completely resolved final assemblies.

### Alignment and SNP analysis

The multiple whole genome alignment of all samples together with 6 complete genomes and 14 genome assemblies from the NCBI database covered 95% of the reference genome *B*. *melitensis* M5-90 and can be assumed to represent the core genome. Choosing strain *B*. *melitensis* 16M (bv1) as reference did not increase the core genome and did not affect the phylogenetic tree. We found significant differences between the two available genome assemblies (GCA_000007125 [Sanger + Primer walking] and GCA_000740415 [Illumina + 454]) of 16M and therefore excluded the assembly based on Sanger sequencing.

**Fig 1:**
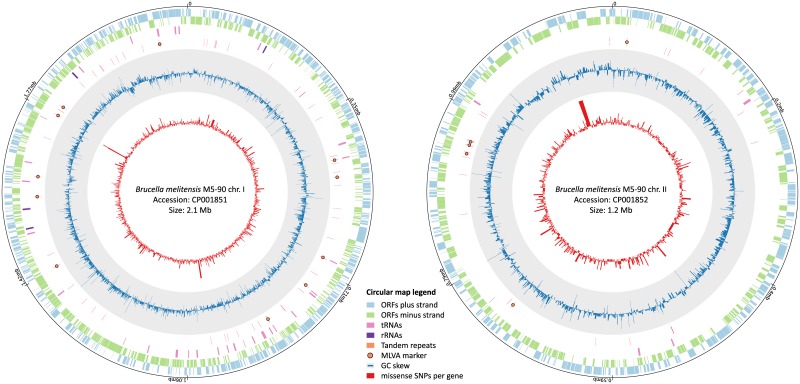
Circular view of the two chromosomes with SNP frequencies and MVLA loci. 5,964 and 3,561 SNPs on chromosome 1 and 2 used in the subsequent analysis are scattered throughout the genome and located, respectively. In contrast, MLVA loci cover only a minor portion of the genome.

The SNP analysis revealed 9,525 filtered core SNPs whereof 5,964 are located on chromosome 1 and 3,561 are on chromosome 2 (excluding those only present in the outgroup, see Fig 1). Out of the 7,752 intragenic SNPs, 2,942 were synonymous mutations, 88 SNPs introduced a premature stop and 4,722 led to amino acid changes (see S1 File). Technical as well as intra-patient replicates were identical according to the core genome SNPs.

**Fig 2:**
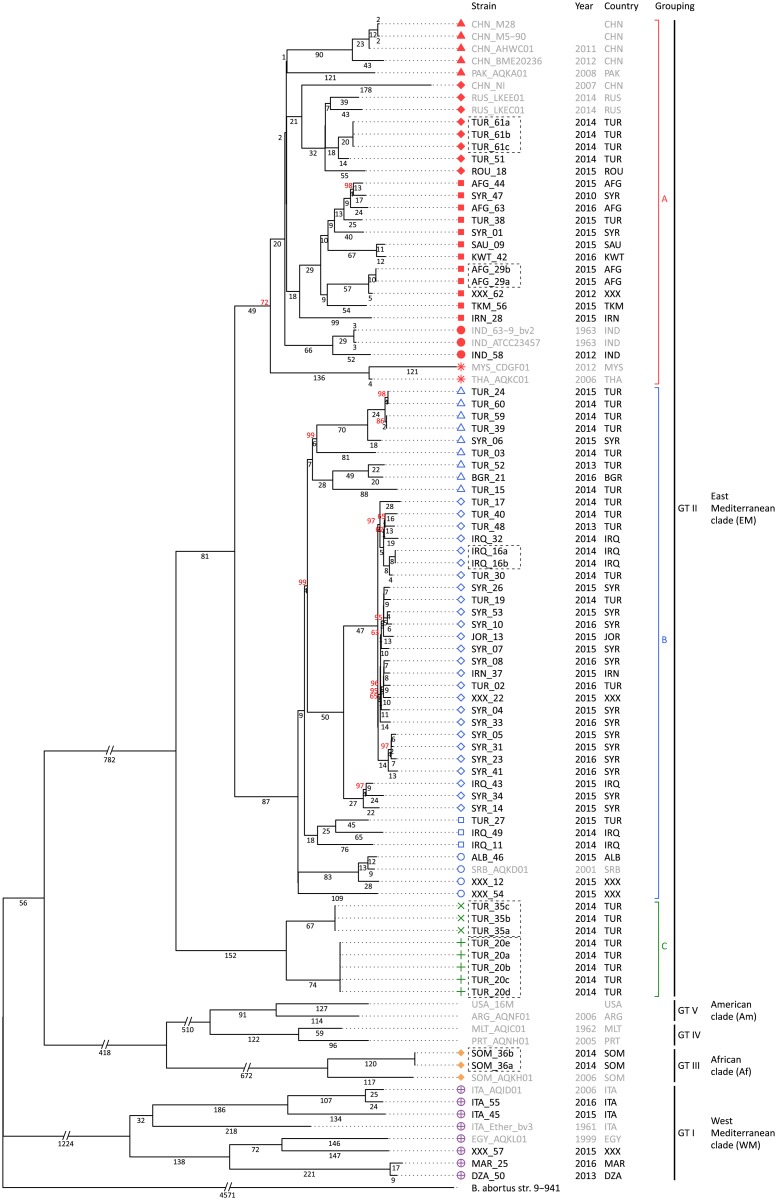
Whole genome SNP-based phylogenetic tree discriminates four major lineages. Clades were inferred using the Maximum Parsimony method. East Mediterranean clade was subdivided in cluster A (red), B (blue), and C (green) with five, four, and two nested clades with unique symbols, respectively. Selected publicly available sequences with geographic attribution were integrated of the dataset, stated with GenBank accession, and marked by grey text colour. Biological or technical replicates were marked with black dotted rectangle. Bootstrap values (based on 1,000 replicates) are shown in red, if less than 100%.

**Fig 3:**
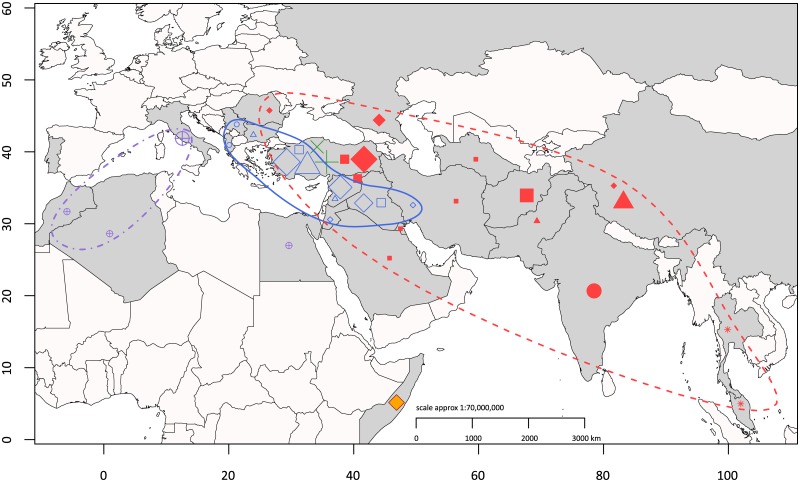
Geographic map of genotypes and probable origin of infection. Genotypes of all patients were coded corresponding to nested clades in Fig 2 and projected to their probable origin of infection on country level.

Phylogenetic analysis based on all whole-genome SNPs revealed spatial clustering (Fig 2). Only 42 SNPs are homoplasious; hence, the consistency index is 0.4%. Five main lineages are clearly distinguished according to Tan et al [18]: Genotype I comprising strains from the Western Mediterranean Region and Egypt, Genotype II with strains from regions of the Eastern Mediterranean Region and the Middle and Far East, as well as Genotype III with strains from the African continent. Genotypes IV and V are assigned to strains from Malta, Portugal and the American continent. Furthermore, several clades and subclades can be discriminated within the lineages and are associated with geographic attributions (see Figs 2 and 3).

Most of the strains isolated from patients in Germany clumped together in the Eastern Mediterranean clade with three distinct subclusters. Subcluster A covered a broad geographic area and could be subdivided in five clades: (i) Far East (China, but also Pakistan), (ii) Southeast Asia (Malaysia, Thailand), (iii) South Asia (India, but also Sudan), (iv) Black Sea area (Turkey, Romania, Russia), (v) Middle East (Iran, Iraq, Turkey, Syria, Turkmenistan, Afghanistan, Kuwait, Saudi Arabia). Subcluster B forms four clades and comprised strains from (i) Turkey-Bulgaria, (ii) Middle East (Syria, Iran, Iraq, Turkey, Jordan) (iii) Turkey-Iraq, and (iv) Southeast Europe (Albania, Serbia). Two of the patients in Germany probably got infected in Turkey and established the third subcluster C.

### In silico PCRs and MLVA

#### bcsp31

As expected, all samples yielded an amplicon of 224 bp size. 3 sequence variants were observed: A dominant genotype with 100 percent conformity of 68 samples, one sample with one SNP variant (52C>T), and 5 samples with another SNP variant (136T>G). The latter demarcated all strains of the Western Mediterranean clade.

#### IS711

The 158 bp multi-copy locus was present in all samples with a median of 9 copies (range 7 to 13). A correlation between copy number and major clade was observed even if the clade specific sample sizes differ from each other: samples from the African and Western Mediterranean clade yielded seven and eight copies, respectively. In contrast, 9 or up to 13 copies of the locus were present in specimen of the Eastern Mediterranean clade.

#### recA

When the analysis was run with *Ochrobactrum*-specific primer sequences (Anth-f/r, Inter-f/r), no amplicon was predicted, whereas genus-specific *Brucella* primers (Bruc-f/r) yielded a 167 bp amplicon with 100% sequence identity for all *B*. *melitensis* samples.

#### BruceLadder PCR and virtual gel electrophoresis

This assay resulted in six identical fragments for each sample. The fragment sizes of 152; 450; 590; 794; 1,071; and 1,682 bps were in accordance with the unique pattern of *B*. *melitensis*.

#### rpoB

The 4,134 bp locus was present in all assembled sequences. 7 SNP variants with 6 different combinations were observed: 1,332A>G; 1,821C>T; 1,886T>C; 2,954C>T; 3,201C>T; 3,747G>A; and 3,927A>G (see Table 2). Interestingly these single nucleotide alterations resolved three major SNP-based lineages, but also four subclades within the Eastern Mediterranean clade. Genetically encoded resistance determinants with phenotypic effect on the susceptibility of rifampin as a commonly used antibiotic for brucellosis treatment were not observed. This was in accordance with phenotypic testing that could not detect rifampin-resistant strains.

**Table 2:** Mutations of *rpoB* gene. SNPs were observed at 7 out of 4,134 positions. rpoB-genotypes consistently matched the WGS clustering.

1332	1821	1886	2954	3201	3747	3927	Clade	Cluster
A	C	T	C	C	G	A	EM	B
A	C	T	T	C	G	A	EM	A
G	C	C	C	C	G	A	EM	C
G	T	C	C	C	G	A	EM	C
A	C	C	C	T	G	G	Af	not assigned
A	C	C	C	C	A	G	WM	not assigned

#### MLVA

**Fig 4:**
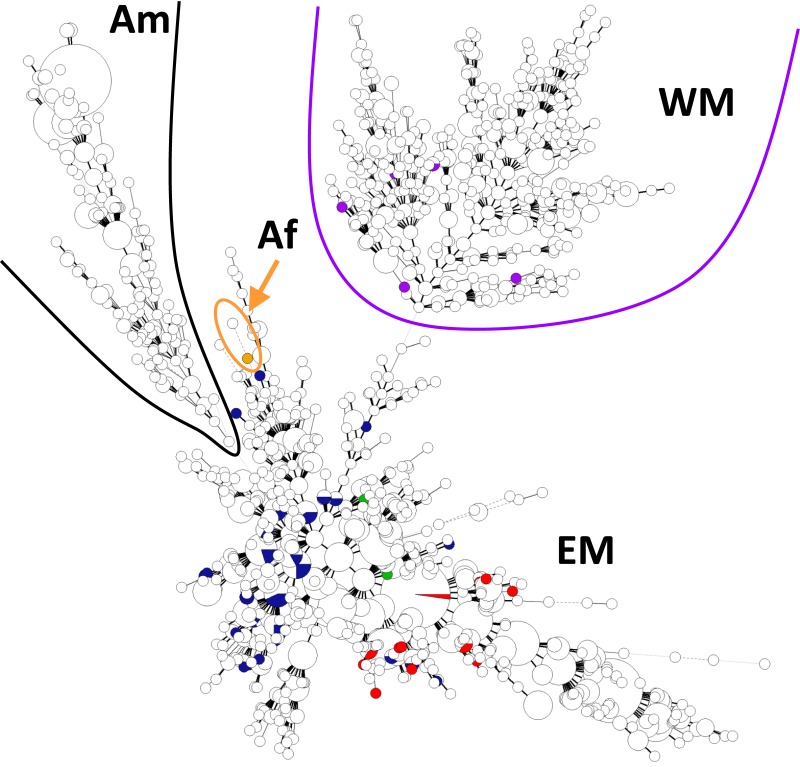
Minimum spanning tree based on MLVA-16 data. Projection of strains used in this study (coloured circles) to a large collection of genotypes from a public MLVA-database. Genotypes identified in this study are dispersed throughout the phylogeny corresponding to multiple geographic origins of infection.

All technical and biological replicates resulted in identical MLVA-16 genotypes in each case. 3 out of 16 loci incurred single-nucleotide substitutions within primer binding sites that could lead to misinterpretation by conventional MLVA (bruce11R 21T>C, bruce43R 10C>T, bruce43R 22C>T, bruce55R 7G>A, bruce55R 13G>A). In total, 52 different genotypes from 63 patients were observed (for details see S1 Table). The public external databases used as reference contained 1,206 different MLVA-16 genotypes of *B*. *melitensis* from 37 countries, if only complete records (full MLVA-16 panel and geographic origin of specimen, at least at country level) are considered (2,177 entries). This study contributes 28 new genotypes, but no new alleles. A MLVA minimum spanning tree analysis was performed with all *B*. *melitensis* entries of the database together with all genotypes found in this study (see Fig 4). The illustration reveals that the genotypes are dispersed throughout the phylogeny corresponding to multiple geographic origins of infection. MLVA-16 led to a similar spatial clustering of isolates compared to the SNP analysis with assigning most of the isolates into the Eastern Mediterranean genetic clade.

## Discussion

The overall incidence of human brucellosis in Europe has dramatically decreased over the past 40 years. The decline can be attributed to the implementation of public health measures including testing and slaughtering of cattle, livestock vaccination, border control and surveillance programmes that largely eradicated the causative agent from the animal reservoir and minimized the risk of transmission to humans. Consequently, a brucellosis-free status was granted by the European Union in most countries of Northern and Western Europe. However, a resurgence of case counts has been observed in Germany since 2014, which may be related to increased international travel and trade, rising flow of migrants and refugees entering Europe, or novel foods like raw camel milk. In order to implement appropriate public health countermeasures, knowledge about the origins of infection and transmission routes associated with the cases would be extremely helpful. Molecular typing of strains is an appropriate tool for the investigation of epidemiological relationships. We, therefore, applied whole genome sequencing and subsequent in-silico SNP-typing to 57 *B*. *melitensis* strains, isolated in Germany from 63 brucellosis patients between 2014 and 2016. Others had already earlier applied the WGS approach to *Brucella*, but primarily just for species discrimination [35,36]. In contrast to such applications, trace-back analysis and the attribution of strains to suspected sources of infection require a high technical discriminatory power. Tan et al. [18] recently published a full genome SNP-based phylogenetic analysis using two own draft genome sequences as well as 49 publicly available genome sequences of *B*. *melitensis* collected globally. However, due to a lack of available sequences from the Middle East at that time, that region had been omitted, although brucellosis is a major public health concern in that area [37]. Azam et al. [38] sequenced and contributed one single isolate of *B*. *melitensis* from a naturally infected goat from India to the public databases. Our study almost doubles the number of publicly available *B*. *melitensis* genomes and also includes isolates from Middle East. This wealth of data will considerably enhance our understanding of the molecular epidemiology of the pathogen.

Our data clearly demonstrate that the increase in brucellosis case numbers in 2014 and 2015 in Germany is associated with strains originating from Middle East. With respect to the limited epidemiological metadata we exclude the possibility that only a few single local sources of infection might have caused this rise. Instead to this, our genetic analysis shows multiple, epidemiological independent strains that obviously had been imported to Germany. This correlates well with the history of most of the patients revealing recent migration from countries where brucellosis is endemic. So, the influx of migrations entering Europe comes along with raised case counts of a hitherto rare disease.

Using a comprehensive collection of strains isolated from patients who acquired brucellosis in various countries, this study also elucidates the distribution of genetic clades, especially of the Eastern Mediterranean lineage, that extends from Turkey to Far East Asia. Whereas strains of subcluster A occur throughout Asia with distinct nested clades in five dedicated areas, strains of subcluster B and C originated only from Middle East and Turkey, respectively (see Fig 3). However, to consolidate our knowledge of the distribution of *Brucella* genotypes, a representative sampling of strains from human and animal cases in endemic countries would be necessary. In addition this might provide new details, as to which degree the observed genetic diversity of the Turkish isolates is confounded by sampling bias.

As compared to Tan et al. [18], we used different SNP-calling parameters. Instead of removing only paralogous genes, we excluded gaps and ambiguous bases as well as mutations located in annotated regions of rRNAs, tRNAs, repeat regions and tandem repeats. We decided to use the more conservative approach to ensure high reproducibility, which is an important requirement for methods that may as well be used for forensic purposes. Consequently, our technical and biological replicates generated identical results. This finding is contradictory to data from Ke et al., who found 5,019 SNPs in derivative strain 16M13w, isolated from BALB/c mice after infection for 13 weeks, compared to strain 16M used to infect the animal model [39]. The whole-genome-based SNP phylogenetic analysis performed by Tan et al. already suggested, that 16M13w may not have originated from China 16M [18]. Our data set comprising *B*. *melitensis* strains isolated from follow-up samples of patients in intervals of 7 days (sample SOM_36) up to 69 days (sample TUR_61), respectively, showed 100% nucleotide identity, which is in line with the low genetic variability of the pathogen and the high reproducibility of the analysis. This strategy may therefore be suitable to differentiate outbreak from non-outbreak strains or a relapse from re-infection. In addition, it is now possible to predict the origin of unknown *B*. *melitensis* samples. Thus, we assume Southeast Europe as origin of infection for sample XXX_12 or the region around Afghanistan and Turkmenistan for sample XXX_62. Naturally, the resolution of the pathogens’ origin is limited to the quality of associated metadata, in other words unclear correlation of routes of migrations and assumed location of infection obscure phylogeographic attribution.

Even though other methods like multilocus sequence typing (MLST) have been published [40], MLVA is actually the most widely used approach for outbreak investigations [41,42] and is considered to be the gold standard of *Brucella* typing [9]. As it is a comparatively inexpensive and reliable method with high discriminating power and a large publicly accessible database is available, it will certainly remain an important epidemiological tool for trace-back analysis of *B*. *melitensis* infections in the future. However, our study provides evidence that WGS achieves a higher resolution [43–45]. MLVA discriminated 52 genotypes from 63 patients, but WGS-based SNP analysis distinguished all patients from each other. At least, the spatial clustering of both methods was largely in agreement. As NGS becomes increasingly available in more and more laboratories, in silico PCRs and in silico MLVA will ensure backward compatibility. MLVA data already acquired in the traditional way will, therefore, remain useful for strain comparison.

As the observed SNPs are scattered throughout both chromosomes, clade-specific variation may pertain to genomic regions that are usually considered as targets for typing or identification assays. Actually, such substitutions were detected in our collection and may be useful to develop real-time PCR assays to discriminate major lineages. Marianelli et al. already suggested to use *rpoB* gene as a molecular marker for the genotyping of *Brucella* species [33]. All four SNPs described by Sayan et al. when applying a *rpoB* SNP assay to a collection of strains isolated in Turkey [46] were also present on the genomes studied here. Moreover, three additional SNPs were observed (1332A>G, 1821C>T, 3201C>T), and the latter delineated the African clade. Our results also indicate that WGS is the method of choice to detect mutations within targets of molecular diagnostic assays, as five mutations within three MLVA loci primer binding sites could be depicted. Furthermore, VNTRs are more susceptible to homoplasy than SNPs in coding regions [43,45].

In summary, our study corroborates WGS to be a suitable tool for trace-back analysis of *B*. *melitensis* suggesting the potential geographic origin of a given strain. The new methodology achieves a higher resolution compared to common typing approaches, but still ensures full backward compatibility. However, the lack of reliable metadata in public databases often hinders an actual resolution below geographic regions or country level and corresponding precise trace-back analysis. Despite the novel role of WGS-derived bacterial typing in discovery genetic relationships, epidemiological investigations (geographic origin and source of infection, transmission route) still are and will be crucial and a requirement, that genomic sequences are meaningful to future investigations. The expansion of WGS and typing databases in the context of clinical microbiology requires the commitment of all contributors to add reliable and comprehensive metadata. Such metadata should also comprise methods used to generate and process the data. In addition, clinicians in Europe should be aware of brucellosis and consider the disease as a differential diagnosis when febrile illness is linked to residence in endemic countries (e.g. travel history, visit of relatives or immigration).

## Supporting information

S1 Table List of MLVA-profiles. This table comprises the determined MLVA-profiles of all samples attached to available epidemiological metadata.
(XLSX)

S1 File Variant calling file for genome comparison. This file contains a list of all observed single point mutations used in the analyses including their annotation using software snpEff.
(TXT)
